# Minced Cartilage in Combination with Autologous Bone Grafting for One-Step Osteochondral Defect Reconstruction in an Athlete's Knee

**DOI:** 10.1155/2021/9501039

**Published:** 2021-11-18

**Authors:** Diane Leyder, Christian Konrads, Patrick Ziegler, Stefan Döbele

**Affiliations:** Department for Trauma and Reconstructive Surgery, BG Klinik, University of Tübingen, Tübingen, Germany

## Abstract

A 17-year-old student athlete suffering from stress-related knee pain asked for help. MRI revealed an unstable osteochondral lesion. Because of time pressure due to the patient's academic exams and his schedule as a basketball player, an autologous chondrocyte transplantation (ACT) as the standard surgical treatment plan was not accepted by the patient. This was mainly because of its two-step character three weeks in between surgeries. Therefore, a surgical one-step therapy option as alternative treatment to ACT was needed. The patient received simultaneous autologous cancellous bone grafting and minced cartilage procedure in a sandwich technique. After successful rehabilitation, the patient continued his studies of sports science and his active career as a basketball player successfully. Several different procedures are used for the treatment of cartilage defects. The following factors play a significant role: defect size, location, patient age, and sports ambitions. In the case described here, ACT would have been the conventional, but not the ideal option in the perspective of this individual patient because of the two-step surgery and the longer rehabilitation time. Therefore, the minced cartilage method presented a valid alternative, even though long-term data are still missing and prospective studies comparing this procedure with others are needed in the future.

## 1. Introduction

Osteochondral lesions of the knee joint are quite often seen in specialized orthopaedic departments. Osteochondrosis dissecans (OCD) affects the male gender 2-3 times more often and has a prevalence of 20-30/100,000 inhabitants [1]. The etiology is not yet fully understood, but currently, there are a multitude of indicators that suggest a mechanically traumatic genesis with repetitive loading, leading to a localized circulatory disturbance in the area of the subchondral bone, which leads to a clustered incidence in athletically active adolescents [2]. The typical location of OCD is the lateral part of the medial femoral condyle. Other areas in the knee joint are affected more seldom, and when they are affected, the prognosis is slightly worse, especially in the femoral trochlea [3, 4]. In up to 40% of the cases, both knees are affected [5].

Different surgical treatment options for osteochondral lesions exist. Autologous chondrocyte transplantation (ACT) is often used and proved to be safe and successful. A disadvantage of this method is that it is a two-step procedure (two surgeries with three weeks in between) with a long period of rehabilitation [6, 7].

We present a case of a young athlete treated successfully using a one-step surgical procedure.

## 2. Case Presentation

A 17-year-old sports student suffering from stress-related pain in the knee joint presented in our outpatient clinic. The pain has been present for about 1 year, mainly during sports activities, but also when walking stairs. Currently, the student is completing his high school diploma and is planning to become a junior basketball player in the first league.

The clinical examination revealed a straight leg axis without malalignment and regular soft tissues. The range of motion was 0/0/130° for extension/flexion, and the medial collateral ligament (MCL) and the lateral collateral ligament (LCL) were stable in full extension and in 30° of flexion. The anterior and posterior drawer tests were negative as was the Lachman test. Furthermore, the meniscus signs were negative and without tenderness over the joint space. Peripheral circulation, motor function, and sensibility were intact.

**Figure 1:**
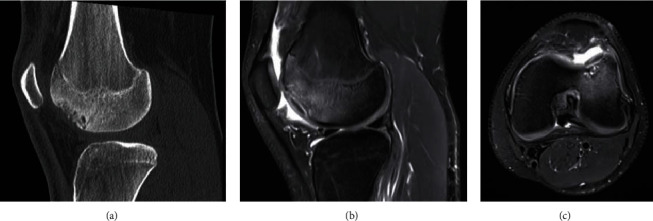
Tomographies of the left knee in a 17-year-old male. (a) CT through the lateral femoral condyle in a sagittal plane showing subchondral cysts. (b, c) MRI in T2-weighted sagittal and axial planes showing subchondral fluid as a sign of dissecate instability.

Magnetic resonance imaging (MRI) revealed subchondral cystic changes at the lateral trochlea with a size of 23 × 18 mm. The cartilage layer appeared intact. Computed tomography (CT) scans showed multiple subchondral cysts (Figure 1). The long leg standing radiograph demonstrated almost straight legs with a valgus of 0.8°. The diagnosis was osteochondrosis dissecans (OCD) stage II-III according to Bruns [8].

With the cartilage layer still intact, an arthroscopy of the left knee joint with cartilage staging and retrograde drilling was performed. The lesion demonstrated to be stable in the probe examination during arthroscopy. This treatment initially led to an improvement in the symptoms. However, when the patient resumed his basketball training after 12 weeks, thereby increasing the weight-bearing, the same complaints as those preoperatively reappeared.

MRI six months after initial retrograde drilling revealed persistent subchondral cysts and increased detachment of the cartilage layer (OCD stage III). Given the persistent pain and the increasing cartilage detachment, all therapeutic options were discussed in detail with the patient and his parents. Because of the defect morphology and size, a two-stage procedure with initial bone augmentation with autologous bone grafting and cartilage cell harvesting with two-stage matrix-associated autologous chondrocyte transplantation (MACT) would be the preferred therapy. However, this was refused by the patient due to the two-stage procedure, which would have led to a prolonged exercise pause. Therefore, a one-stage procedure with autologous bone augmentation from the tibial head and autologous chondrocyte transplantation using minced cartilage (AutoCart, Fa. Arthrex) was carried out instead.

**Figure 2:**
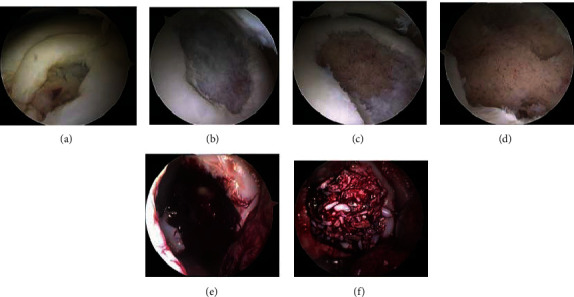
Surgical treatment of an osteochondral lesion by autologous bone grafting and minced cartilage. (a) Osteochondral lesion with (b) subsequent cartilage removal by (c) debridement with a curette and (d) removal of the subchondral cysts. (e) View of the defect after miniarthrotomy and (f) after autologous cancellous bone grafting and application of the minced cartilage.

Surgery started with arthroscopy, which revealed an unstable osteochondral defect of 20 × 15 mm. The unstable cartilage remnants were reprocessed with the minced cartilage instrumentation. The underlying avital cancellous bone with cystic changes was completely debrided with a curette and a sharp spoon. An anterolateral miniarthrotomy with a skin incision of 3 cm was performed to insert the cancellous bone, which was harvested from the anterolateral tibial head. Then, the prepared minced cartilage was applicated, and fixation was secured using platelet-rich plasma and fibrin. In this way, the osteochondral defect was completely filled (Figure 2). It was of importance not to overfill the defect, thereby creating a prominent surface. The stratum synoviale of the joint capsule was closed using PDS suture, and the stratum fibrosum was closed using vicryl. Then, the skin incision was closed.

The rehabilitation protocol consisted of partial weight-bearing for 8 weeks postoperatively, followed by muscle strengthening and coordination training. The patient was back to basketball four months postoperatively and was pain free demonstrating full range of motion at the last follow-up examinations six and twelve months postoperatively.

## 3. Discussion

This case demonstrated successful one-step treatment of an osteochondral lesion using autologous bone grafting and minced cartilage in a sandwich technique.

In OCD stage II, when conservative therapy fails, the treatment recommendation is local revascularization. This is accomplished by antegrade or retrograde drilling to achieve reossification in the subchondral region [9].

For OCD stage III, there are several surgical options. There is the possibility of bone stimulation in the form of microfracturing or nanofracturing to induce the formation of fibrocartilage, which can be performed up to a defect size of 4 cm^2^. The short-term results of this treatment are satisfactory. Microfracturing can also be performed as part of autologous matrix-induced chondrogenesis (AMIC) with the application of a biological membrane, which can improve the long-term results [10].

Autologous osteochondral mosaicplasty can also be performed up to a defect size of 4 cm^2^, but ideally, the defect should be smaller than 2 cm^2^. For this method, positive long-term results were documented after an 8-year follow-up. For larger defects, also the morbidity at the harvesting site for osteochondral transplantation must be taken into account and may be considered a limiting factor [11].

In patients with a larger defect, autologous chondrocyte transplantation (ACT), with positive 5-year results, is currently the leading standard. However, this is a two-stage procedure with additionally high costs [12].

The minced cartilage procedure is a relatively simple and cost-effective technique to transplant autologous cartilage fragments in a single-stage procedure. Minced cartilage has a high biological healing potential because it is composed of autologous hyaline cartilage cells. It can be used for both minor and major cartilage defects and osteochondral lesions. Currently, however, there is a lack of long-term data to evaluate the therapeutic benefits [13]. Preliminary data show a satisfactory outcome at 2 years in a relatively small number of patients. Long-term data from a larger patient population is still missing [14].

We want to use this case not only to report the successful application of the described one-step surgical procedure using minced cartilage demonstrating excellent short-term clinical outcome but also to emphasize the importance to always treat the subchondral bone, as far as it is compromised, and not only treat the cartilage in those cases.

## 4. Conclusion

Several different procedures are used for the treatment of cartilage defects. The following factors play a significant role: defect size, location, patient age, and sports ambitions. In the case described here, ACT would have been the conventional, but not the ideal option in the perspective of this individual patient because of the two-step surgery and the longer regeneration time. Therefore, the minced cartilage method presented a valid alternative, even though long-term data are still missing and prospective studies comparing this procedure with others are needed.
